# Prospective Monitoring of Circulating Epithelial Tumor Cells (CETC) Reveals Changes in Gene Expression during Adjuvant Radiotherapy of Breast Cancer Patients

**DOI:** 10.3390/curroncol28050302

**Published:** 2021-09-08

**Authors:** Matthias Mäurer, Katharina Pachmann, Thomas Wendt, Dorothea Schott, Andrea Wittig

**Affiliations:** 1 Department of Radiotherapy and Radiation Oncology, University Hospital Jena, Bachstraße 18, 07743 Jena, Germany; thomas.wendt@med.uni-jena.de (T.W.); andrea.wittig@med.uni-jena.de (A.W.); 2 Transfusion Center Bayreuth, Kurpromenade 2, 95448 Bayreuth, Germany; kpachmann@laborpachmann.de (K.P.); dschott@simfo.de (D.S.)

**Keywords:** circulating tumor cells, gene expression analysis, radiotherapy, early-stage breast cancer, prognostic marker

## Abstract

Circulating epithelial tumor cells (CETC) are considered to be responsible for the formation of metastases. Therefore, their importance as prognostic and/or predictive markers in breast cancer is being intensively investigated. Here, the reliability of single cell expression analyses in isolated and collected CETC from whole blood samples of patients with early-stage breast cancer before and after radiotherapy (RT) using the maintrac^®^ method was investigated. Single-cell expression analyses were performed with qRT-PCR on a panel of selected genes: GAPDH, EpCAM, NANOG, Bcl-2, TLR 4, COX-2, PIK3CA, Her-2/neu, Vimentin, c-Met, Ki-67. In all patients, viable CETC were detected prior to and at the end of radiotherapy. In 7 of the 9 (77.8%) subjects examined, the CETC number at the end of the radiotherapy series was higher than before. The majority of genes analyzed showed increased expression after completion of radiotherapy compared to baseline. Procedures and methods used in this pilot study proved to be feasible. The method is suitable for further investigation of the underlying molecular biological mechanisms occurring in cells surviving radiotherapy and possibly the development of radiation resistance.

## 1. Introduction

In breast cancer patients, adjuvant radiotherapy has been shown to reduce not only the risk of locoregional recurrence but also the risk of distant metastases, thus to reducing breast cancer mortality (Early Breast Cancer Trialists’ Collaborative, Darby [1]). Adjuvant irradiation destroys subclinical disease in the breast and possibly in locoregional lymph nodes, which is believed to prevent seeding of tumor cells from persistent reservoirs of locoregional disease and thus avoid metastasis formation.

Tumor cells circulating in the bloodstream (CTC) are considered to be responsible for the formation of metastases [2,3]. Therefore, their importance as prognostic and/or predictive markers in breast cancer is being intensively investigated [4,5,6,7,8,9,10].

The determination of circulating epithelial tumor cells (CETC) promises real-time monitoring of treatment effects—including effects of radiotherapy—as well as longitudinal monitoring of treatment response and early detection of tumor relapse [11]. In patients with metastatic breast cancer, CTC have been proposed as prognostic biomarkers [12,13]. To date, the method has, however, not been implemented in routine clinical practice as a basis to guide treatment decisions, which is partly due to a lack of standardization in analysis approaches [14,15]. 

Next to the number of CETC, the molecular characterization of CETCs is crucial to relate molecular characteristics of individual CETC to molecular characteristics of the primary tumor, but also to follow the fate of circulating tumor cells during treatment. Equally important is the description of relevant genetic changes that may be caused by treatment-related effects in CETC. However, due to the relative rarity and heterogeneity of circulating tumor cells, their isolation and characterization at the molecular and genetic level is challenging and technically difficult [16,17,18]. 

In this study the maintrac^®^ method was used to analyze changes in the number of CETC before and after adjuvant radiotherapy in a group of prospectively enrolled patients with breast cancer. The study further investigated changes of molecular characteristics and expression of a panel of selected genes of isolated CETCs before and after the radiotherapy series. The study demonstrates the reproducibility of the method for single cell expression profiling during monitoring of the influence of radiotherapy in preparation of a larger study that will allow inclusion of a higher number of patients.

## 2. Materials and Methods

The study is designed as a biology-driven translational trial to investigate the feasibility of isolating and collecting circulating epithelial tumor cells from whole blood samples before and after radiotherapy using the maintrac^®^ method, and to analyze possible up- and/or down-regulation of a panel of selected cancer-related genes. 

### 2.1. Inclusion Criteria

Patients with histologically-proven, primarily non-metastasized breast cancer were eligible if adjuvant radiotherapy after breast-conserving surgery was planned. Other eligibility criteria were age ≥ 18 y, WHO performance status ≤ 2, no severe concomitant disease, and in particular no additional tumor diseases. There were no restrictions with respect to preceding systemic treatments like (neo)adjuvant cytostatic or antihormonal drugs if indicated according to current guidelines. All patients gave informed consent prior inclusion in the study. The trial was approved by the Ethics Committee of the University Hospital Jena (1 September 2012) under No. 0921-08/02 and is registered (2 May 2019) at trials.gov under NCT03935802.

### 2.2. Study Procedures

Routine clinical parameters were prospectively collected, including baseline history, breast cancer specific procedures and treatments, as well as radiotherapy. Patients were prospectively followed for local control, metastasis-free survival, and overall survival for a minimum of 5 years after diagnosis. 

### 2.3. Radiotherapy

All patients received adjuvant radiotherapy according to current guidelines [19]. The breast and chest wall were irradiated with a 3D-conformal technique using tangential opposing fields to a total absorbed dose of 50.0 Gy in 25 fractions. In cases where a boost was indicated, the tumor bed received an additional dose of 16.0 Gy in 8 fractions. 

### 2.4. Sample Processing and Analysis

Blood samples were drawn as part of routine laboratory checks on the first and the last day of radiotherapy, where 10 mL blood (EDTA-tube, Sigma-Aldrich Co., Munich, Germany) were additionally taken for analysis within this trial. 

**Figure 1:**
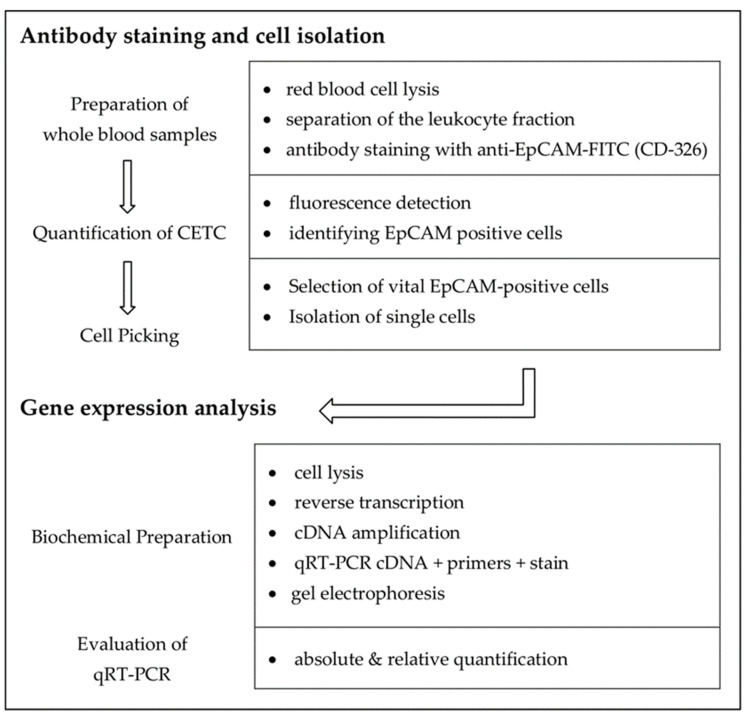
Workflow of antibody staining, cell isolation, and determination of mRNA contents.

Samples were processed for picking viable, epithelial cell adhesion molecule (EpCAM)-positive cells and analysis of selected messenger ribonucleic acids (mRNAs), which are typically found in breast cancer cells, as detailed in Figure 1. 

Each sample was analyzed with respect to:

- quantitative immunofluorescence detection of viable CETC and
- quantitative gene expression analysis of single CETC by qRT-PCR to analyze for differential expression of selected genes.

### 2.5. Immunofluorescence Assay and Quantification

Isolation of individual cells from whole blood was carried out by employing a method previously described by Pachmann et al. [11,17,20]. Briefly, 1 mL whole blood drawn into Sarstedt EDTA tubes (SARSTEDT AG & Co., Nümbrecht, Germany) for anti-coagulation was mixed with 10 mL lysis buffer (QIAGEN, Hilden, Germany) for erythrocyte lysis followed by centrifugation at 300 rcf to separate the cellular fraction from the plasma fraction of blood samples. The cellular fraction was re-suspended in 500 µL phosphate buffered salt solution (QIAGEN, Hilden, Germany) and incubated with 20 µL of master mix (120 µL EpCAM-FITC, 100 µL 10% BSA, 4 µL 7AAD (500 µg/mL), and 776 µL PBS-EDTA) (fluoroisothiocyanate (FITC)-conjugated anti-epithelial cell adhesion molecule (EpCAM) antibody (CD-326, Miltenyi, Bergisch Gladbach, Germany) and 7-Aminoactinomycin D (7AAD, Sigma-Aldrich Co., Taufkirchen, Germany)). Finally, 20 μL of this suspension were transferred to a 96-well plate (Eppendorf twin.tec PCR plate 96, Eppendorf AG, Hamburg, Germany) and mixed with a PE-formalin solution (10% Formalin solution and PBS-EDTA 1:1).

After 1 h of sedimentation, immunofluorescence detection was initiated using a microscope-based imaging platform for fully automated image acquisition (ScanR Olympus IX81 ZDC, Olympus GmbH Hamburg, Germany). Each well was scanned by taking 94 individual images using a 20×-objective and analyzed for the presence of EpCAM-positive cells. Due to the different emission intensities of the markers (515–545 nm for FITC and 635–655 nm for 7AAD), objects with exclusively EpCAM-positive surface staining (viable cells) and combined EpCAM- and 7AAD-stained avital cells could be automatically detected. In addition to the automatic detection and classification (viable vs. nonviable) of cells, up to 100 EpCAM-positive cells per sample were controlled visually in a randomized fashion for accuracy of classification. From the number of viable EpCAM-positive cells detected per well, the number of cells/mL was calculated. 

We additionally examined blood samples of 9 healthy subjects without tumor disease as a control group. In none of these subjects, EpCAM-positive cells were detected.

### 2.6. Single Cell Isolation and Quantitative Real-Time PCR

**Figure 2:**
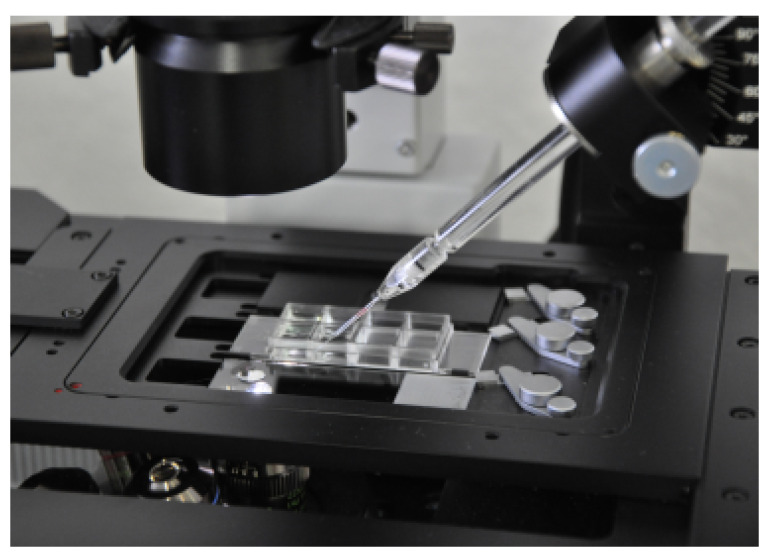
High-performance stereomicroscope and preparation platform MMI CellEctor plus™ with a three-dimensionally-controllable micromanipulator.

**Figure 3:**
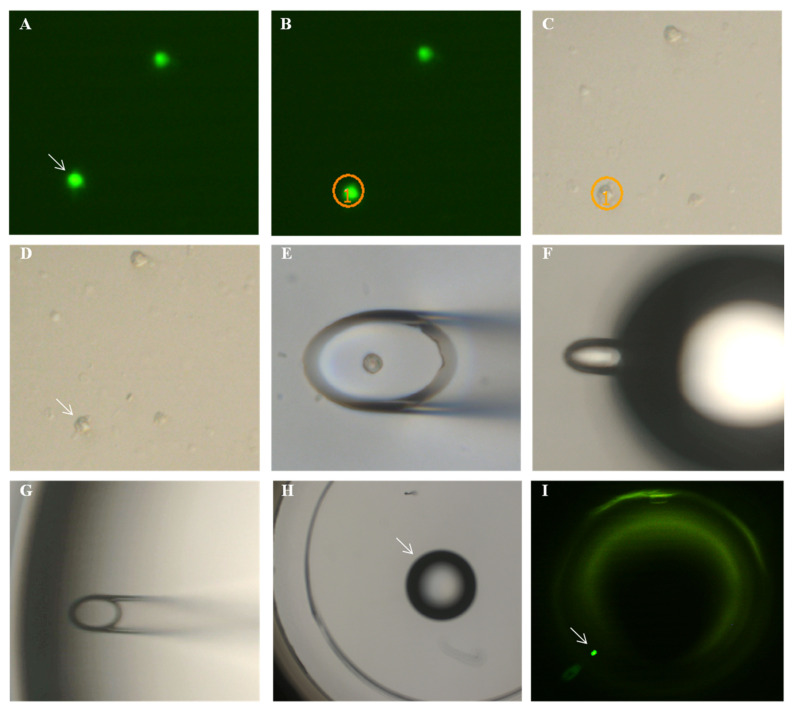
Steps of micromanipulation: (**A**) the cell to be picked is located in fluorescent light and (**B**) is circled by the software. (**C**,**D**) The cell is located in transmitted light. (**E**) The opening of the capillary is positioned over the cell and aspirates the cell. (**F**) The droplet with the cell is blown out from the capillary (diameter 25 µm), and (**G**) it is confirmed that the cell is no longer inside the capillary but that it is released with the (**H**) droplet and can be detected (**I**) in fluorescent light.

From an aliquot of the above stained cells, single EpCAM-positive cells were separated from other white blood cells using a semi-automated fluorescence microscope (Olympus, Hamburg, Germany). A drop of cell suspension containing cells of possible tumor origin was placed on a microscope slide, and EpCAM-positive cells were detected under the microscope (Figure 2 and Figure 3). To only collect viable cells, cells with morphologically intact cell membrane and nucleus as well as an intense EpCAM signal were selected. The cell suspension was prepared to allow aspiration of only one cell into the capillary. Selected cells were individually aspirated semi-manually with an MMI CellEctor (MMI, Eching, Germany) into 100 nL buffer solution into a glass capillary and transferred into a 100 µL PCR cup, making sure that only this one cell was deposited before being stored individually at −18 °C.

Cell lysis and subsequent cDNA amplification were performed using the whole transcriptome amplification kit according to protocol A of the manufacturer (CellAmp™ Whole Transcriptome amplification kit (Real Time) TaKaRa Bio Inc., Otsu, Japan). cDNA amplification was performed in 21 cycles using a Mastercycler^®^ (Eppendorf realplex4, Mastercycler eppgradient S, Eppendorf AG, Hamburg, Germany). The resulting 25 µL of cDNA amplification reaction mix was stored at −20 °C. 

Subsequently, quantitative real-time PCR (qRT-PCR) was performed in 96 well plates using the LightCyclerR 480 SYBRGreen 1 Master Kit (Roche Diagnostics GmbH, Mannheim, Germany). A known concentration of beta-actin was used in control reactions to calculate absolute values [21]. Following the manufacturer´s instructions, 1 µL of amplified cDNA was pipetted into wells of a 96-well plate, 19 µL of master mix were added, and the sample was cooled to 4 °C. Amplified cDNA from one cell was tested for specific mRNAs in eight separate wells containing one primer pair each. qRT-PCR reactions were performed for 40 cycles. A melting curve was generated for quality control. Absolute quantification and analysis of melting curves were undertaken using the qRT-PCR machine’s software package (Eppendorf realplex4, Mastercycler eppgradient S, Mastercycler ep realplex 2.0, Eppendorf AG, Hamburg, Germany). The resulting gene product was verified by gel electrophoresis. Sequences of the 11 selected primer pairs are summarized in Table 1. All primers were produced individually (Jena Bioscience GmbH, Jena, Germany). Negative control experiments were undertaken using 1 µL RNase-free water.

### 2.7. Statistical Analysis

Single cell analysis is associated with variability of the measured values. The mRNA amount of glyceraldehyde-3-phosphate dehydrogenase (GAPDH) in our study showed a large variability between the single cells as expected. Due to the biology of cellular transcription, data normalization using housekeeping genes is not meaningful when quantifying single cells [22]. The absolute values of the cDNA concentrations of the 8 cells per patient as well as the measurement time points were mapped as absolute values. The quotient was then calculated from the values of the 8 cells collected prior to and after radiotherapy for all genes serving as a measure of gene activity and compared to the mean values. 

In all examined cells, GAPDH-mRNA could be detected with at least 7.20 copies/μL. This result confirmed selected CETC to be viable cells. We used the SPSS21 software package (IBM Statistics, SPSS Inc., New York, NY, USA) for comparative statistics. 

## 3. Results

Nine eligible patients were included in the trial between September 2011 and September 2012 [23]. All but one patient exhibited estrogen receptor positive tumors and had already received endocrine treatment during the period of radiotherapy. Estrogen receptor positivity was not determined in the circulating tumor cells. The mean follow-up was in 88 months (38 months–100 months). During follow-up, two patients experienced a second tumor diagnosis: patient 2 was diagnosed with colon carcinoma 84 months after radiotherapy, patient 5 suffered from a local relapse, and patient 6 was diagnosed with malignant melanoma 17 months after radiotherapy for breast cancer. All patients were alive at the time of the last visit. Patient characteristics are summarized in Table 2.

### 3.1. Changes in CETC Number

In all patients, viable CETC were detected prior to and at the end of radiotherapy (detection rate: 100%) and the cell number was determined. Blood samples from nine healthy subjects, who volunteered as a control group without tumor disease, were additionally examined. In none of these subjects, EpCAM-positive cells were detected.

In seven of the nine (77.8%) subjects investigated, the CETC number was higher in the blood sample drawn at the end of the radiotherapy series as compared to the CETC number prior to the start of radiotherapy (Table 3). Statistical evaluation showed a significantly increased number of viable CETC at the end of the radiotherapy series compared to the CETC number before start of radiotherapy (*p* = 0.009). The fold increase (quotient) was moderate as compared to that observed in patients with primary breast cancer under adjuvant chemotherapy (6). 

### 3.2. Gene Expression Profiles of Single Circulating Epithelial Cells

In two patients, isolation of single cells was not possible due to logistical reasons. In the remaining seven patients, a maximum of eight vital CETC per patient were picked prior to as well as after the end of the radiotherapy series for gene expression analysis. As shown in the materials and methods section, the approach for picking individual cells ensured that only the targeted cell was aspirated and deposited. Previous studies showed that cells isolated in this way carry the same mutations as their corresponding primary tumor and also express its tumor tissue characteristics [17,24].

The expression of a panel of selected genes, which encode for proteins involved in processes necessary for metastatic spread, such as proliferation, differentiation, migration, adhesion, and apoptosis, were analyzed in these individually isolated cells. A total of 1505 individual gene copy numbers were measured (copy number > 0/μL) from 131 EpCAM-positive cells most probably of tumor origin. Each individual cell could be assigned a defined copy number of the respective gene.

A typical example of the heterogeneity in upregulation of gene expression of 11 genes in eight cells from Pat. ID 2 is shown in Table 4. In some cells, several genes were highly upregulated, e.g., cell 6: GAPDH, BCL-2, Cox2, PIK3CA, and TLR 4 whereas EpCAM, NANOG, Her2, Vimentin c-Met, and Ki-67 were only moderately upregulated. In cell 7, GAPDH, Cox-2, and TLR 4 were strikingly highly upregulated.

Relative quantification showed that patients exhibiting an increased cell number under radiotherapy also showed overexpression of almost all genes investigated (Table 5). The two patients without an increase in CETC numbers (Pat. ID 4 and ID 5) showed the lowest relative gene expression apart from NANOG in patient ID 4. The extent of increase of expression in selected genes differed considerably between patients. 

A decrease of expression for several genes was observed only in one patient whose CETC number decreased after radiotherapy (Pat. ID 4) in comparison to the CETC number prior to radiotherapy. 

Since the number of copies in patients ID 2 and ID 4 varied particularly strongly for some genes (especially BCL2, HER2, Vimentin), they were censored in the statistical analysis. Considering the extreme values, a symmetrical distribution of the mean values could be achieved (Shapiro–Wilk test: *p* ≤ 0.05, boxplot diagram of the mean value difference symmetrical). It was possible to use the T-test for connected samples. 

**Figure 4:**
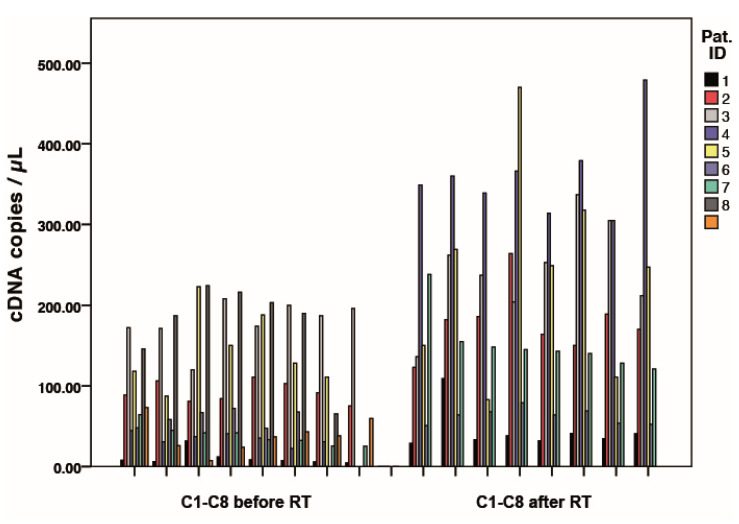
Results of NANOG expression analysis for eight single cells (C1-8) before and after radiotherapy for all patients. Each bar represents the expression of a single tumor cell/patient.

The different expression level of the single cells is shown in the example of NANOG for all patients in Figure 4. The variability of expression between individual cells in the patients is shown. The copy number amplitudes are higher after RT, indicating an increase in gene expression. 

**Figure 5:**
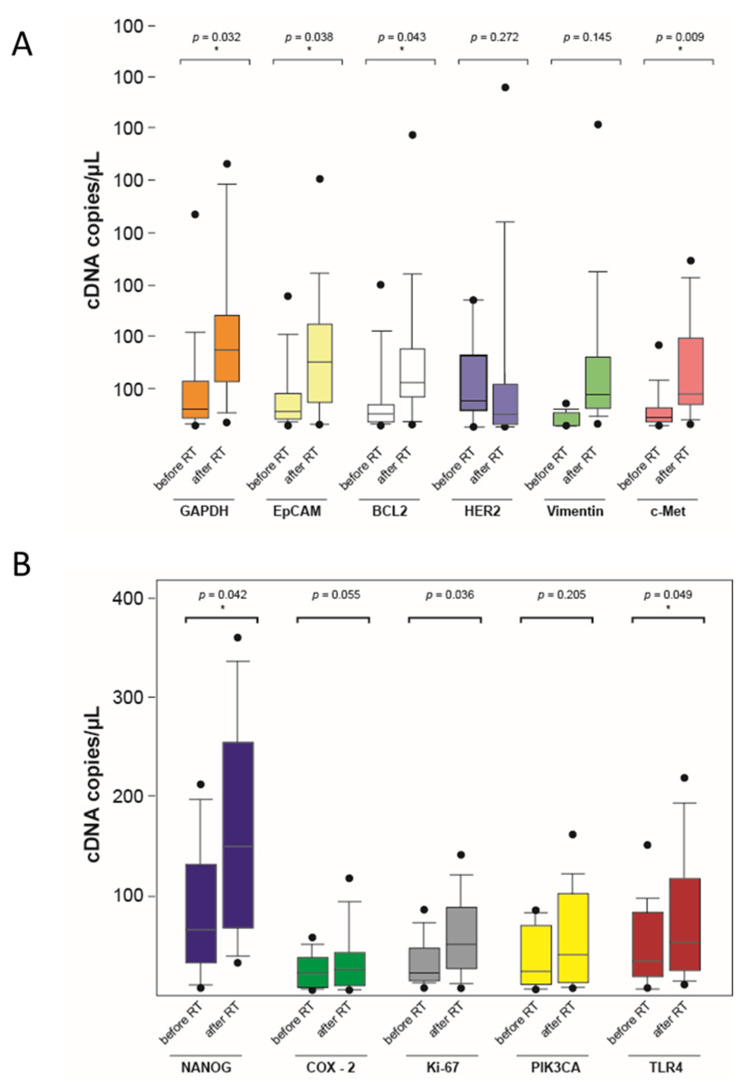
Mean mRNA copy number before and after the end of radiotherapy in a boxplot diagram (* significant, *p* < 0.05) (**A**,**B**).

The distribution of the mRNA copies for each gene is shown below in the box plot diagrams (Figure 5). Although the measured values varied, the mean values of the mRNA copy numbers are higher after irradiation in all the genes investigated. Except for PIK3CA, ERBB2, and Vimentin, statistically significant changes in gene activity could be detected. 

All results of the RT-PCR were verified by gel-electrophoresis where the corresponding gene product could be detected.

## 4. Discussion

Potential changes of the tumor cell activation during the course of a disease render longitudinal analysis of circulating tumor cells promising for a more complete understanding of the complex processes underlying treatment response or relapse. Consequently, CETC numbers and their relevance as prognostic or predictive factors were investigated in numerous studies [4,11,25,26]. Further characterization of CETC on a molecular and genetic level may reveal their potential role as an indicator of response to treatment [27,28,29]. Molecular characterization of CETC is, however, technically challenging as is their unequivocal mapping with cells of the primary tumor or even metastases. Hence, following the fate of tumor (sub-)clones during the course of disease is not currently possible [30].

Therefore, in this study, radiation-related changes in CETC numbers after curative breast-conserving surgery and alterations in expression of selected genes in individual EpCAM-positive CETC were prospectively investigated using the maintrac^®^ method. 

Since this was a feasibility study, both the number of patients included and the number of cells studied were considered sufficient. The number of patients included so far allows a first description of the observed effects, but is not high enough for definite conclusions. Confounding factors could influence CETC counts and gene expression profiles in addition to radiotherapy.

### 4.1. Comparison of Methods for Detection of CETC

Different approaches exist for the identification and characterization of circulating tumor cells, where genetic and immunological methods need to be distinguished. A frequently used enrichment method is immunomagnetic separation using the CellSearch™ system. It has been approved by the FDA as a tool for the detection of CTC in metastatic breast cancer [31,32,33]. The CellSearch™ system variably defines a threshold of 1–5 cells/7.5 mL blood (≥0.67 cells/mL blood) in patients with primary or metastasized tumors as a sign of poor prognosis [34,35].

In contrast to the method used in this work, the CellSearch™ system aspirates the blood into special CellSave tubes with additional fixation solution instead of standard EDTA monovettes. The fixation solution in the CellSave tubes seems to strongly influence the detectable amount of circulating tumor cells. It has been shown that the use of CellSave tubes reduces the detection of EpCAM-positive cells by more than 10-fold compared to the maintrac^®^ method. According to other studies, a high proportion of cell fragments is detected using the CellSearch™ method, which is, however, attributed the same prognostic significance as intact, vital cells [36].

In our study using the maintrac^®^ method, an approach omitting fixation and enrichment steps, the number of EpCAM-positive cells was much higher with 1220 and 18,070 cells/mL blood in clinically non-metastatic women. This included cells with very low EpCAM expression, which may be lost by enrichment steps. In spite of confirmed metastatic breast cancer, the CellSearch™ method detects circulating tumor cells in only 10–60% of cases in blood or bone marrow [37]. The maintrac^®^ method detects CETC in 90% of cases of primary non-metastatic breast cancer and an increase in these numbers during therapy is highly significantly correlated with relapse [11].

The number of circulating tumor cells determined by various methods is controversially discussed [38]. Given the heterogeneous results concerning the number of detected circulating tumor cells with various methods of analysis, standardization of test methods is of utmost importance [39]. The high number of CETC detected with the method applied here is advantageous with respect to micromanipulation for isolation of individual cells in patients with early breast cancer [17,29,40] to analyze intra-individual changes in CETC numbers during the course of treatment and to amplify mRNA from the individually isolated cells.

The possibility of false positive results in light of the high detection rate must be considered. However, the fact that mRNA of the candidate genes could be amplified from the individually isolated cells suggests that these cells are viable.

### 4.2. Expression Pattern of EpCAM

The EpCAM antigen used in the maintrac^®^ process is not exclusively expressed on the surface of breast cancer cells, but also on normal epithelial cells, e.g., of the gut and the lung. EpCAM overexpression in breast cancer correlates with tumor mass, lymph node status, and the presence of estrogen receptors [41]. The EpCAM antigen is also found in other cancers such as lung or colorectal carcinoma [42,43,44]. Therefore, the detected cells comprise circulating tumor cells but the presence of additional nonmalignant cells cannot be completely excluded. EpCAM-positive cells are usually not detectable in healthy subjects, as confirmed in our study in blood samples from healthy subjects.

By labeling the CETC with EpCAM and subsequently picking EpCAM-positive cells, PCR results could be assigned exclusively to the respective cells. It has been assumed in the past that the detection of tissue-specific mRNA in peripheral blood indicates the presence of circulating tumor cells [45,46]. However, irrespective of the enrichment method used, samples are contaminated with white blood cells, resulting in reduced specificity, especially in the analysis of mixed populations of cells allowing only for cross-sectional information. In contrast, our analysis of individual cells displays the heterogeneity of the circulating tumor cells. Since mRNA is transcribed only in viable cells and degraded very rapidly during cell decay, the combination of upstream individual cell isolation and subsequent multiplex PCR using single cells in this study minimized these sources of error and increased sensitivity and specificity [7,18,47].

### 4.3. Quantitative Polymerase Chain Reaction

With regard to the qRT-PCR results, difficulties arise especially with genes which are expressed at low levels. Intercellular differences in gene expression can be large, and extreme values of individual cells can largely influence the mean value [48]. As expected, the number of gene copies detected in single cells in this study varied considerably between undetectable (HER2 for Pat. ID 1) and 379.0 copies/µL (NANOG for Pat. ID 4). This led to large standard deviations.

Nonetheless, the averaged gene copy numbers in up to eight analyzed single cells was significantly increased for most genes after irradiation in comparison to the average copy number in cells before irradiation.

In this study, analysis of single circulating cells by qRT-PCR allowed for discerning heterogeneities in gene activity between individual CETC of a patient, and additionally determined changes in gene activation during therapy to provide a starting point for in-depth research of a higher number of patients.

### 4.4. Quantitative Measurement of the CETC

In seven of the nine patients (77.8%) the number of CETC was higher after radiotherapy as compared to the CETC number before start of radiotherapy. On average, a doubling of CETC numbers was observed. One possible explanation for this observation is the release of cells from occult residues, which subsequently enter the microenvironment and the bloodstream [49,50,51]. These could represent cells that have survived radiation and may be a sign of radioresistant cell clones already present prior to the radiotherapy series [52]. The changes in the local and systemic environment, such as inflammation induced by radiotherapy, may stimulate the expansion of the cells that are circulating in blood [53]. It is controversially discussed whether the cells found in blood are derived directly from the primary tumor [54,55,56]. Further investigations including analysis of molecular markers of the CETC at serial time points during treatment are necessary to clarify this question.

### 4.5. Gene Expression Analysis

In patients with increased CETC numbers after radiotherapy, we also found an increased expression of the majority of genes in single CETC of the respective patients.

The examined genes encode proteins involved in cell metabolism, but also in inflammatory and immunological processes, cell proliferation, adhesion, differentiation, and migration as well as angiogenesis, apoptosis inhibition, and preservation of cellular pluripotency [57,58,59,60,61,62,63,64,65,66,67].

It is known that irradiation causes dose-dependent effects, leading to DNA damage by direct or indirect ionization and additionally by generating reactive oxygen species (ROS), thereby destroying cancer cells. Ionizing irradiation can however also promote epithelial-mesenchymal transition, angiogenesis, invasion, and finally metastasis formation [68,69,70,71,72,73]. As an example, PI3K/Akt/mTOR signaling regulates cell growth and proliferation apoptosis and DNA damage response but also acts as a main driver of cellular survival mechanisms after irradiation. In addition, ionizing radiation increases Akt phosphorylation in numerous tumor entities, possibly causing resistance to therapy. The gene activation after radiotherapy observed in this trial is therefore is not surprising, however its relevance and underlying molecular mechanisms are unclear. Despite the small patient and cell numbers explored in this study as well as the variability of the measured values, differences between pre-radiation and post-radiation cells were significant in 5 out of 11 genes over all patients. It might well be that effects mediated by radiation therapy itself could be responsible for the upregulation of gene expression.

In addition to direct impact ionization at the cellular level, immunomodulatory phenomena are known which are dose-, time-, and volume-dependent and can occur both locally and systemically [74,75]. Radiotherapy leads to cell death in some of the cells, thereby evoking phagocytosis of these cells and again inducing an immune response. This particularly underlines the activation of TLR4 and COX-2 observed in this study, suggesting that increased cytokine and chemokine production suppresses an adaptive immune response and promotes metastatic processes [76,77]. It is also possible that the detected CETC are radio-resistant tumor stem cells or subclones with pronounced differentiation potential, which can survive treatment (especially if such cells circulating with the blood stream do not receive the total radiation dose applied locally to the breast/chest wall) and lead to a selected subpopulation of CETC with increased malignant potential [78,79,80,81,82,83,84,85,86,87,88]. The strong activation of NANOG in all patients seems to support this hypothesis [82]. With regard to the genes described, these surviving cells would then represent a particular risk for the development of distant metastases [89].

Based on the present pilot study, further investigations are currently being performed on a larger number of patients and single cells in order to investigate the molecular biological and functional effects of radiotherapy on CETC in more detail. In order to identify prognostic and predictive biomarkers, a prospective follow-up study of the group will investigate the impact of radiotherapy on CETC, its gene expression profile, and immunophenotype over the course of treatment including follow-up (study number NCT03935802).

### 4.6. Influence of Endocrine Therapy

Endocrine therapy might influence changes in the numbers of CETCs as well as gene expression as a confounding factor [87]. This and other confounders cannot be eliminated as patients were treated according to current guidelines. In all examined patients in this study, endocrine therapy was started already prior to RT.

Data on the effect of endocrine treatments on CETC number are scarce. It is known that patients whose CETC count increases after endocrine therapy are significantly more likely to suffer a relapse [90]. However, to what extent endocrine therapy has a direct influence on the single CETCs is not clear so far.

### 4.7. Clinical Follow-Up

The only patient who suffered a local relapse within five years after RT was patient ID 5. The number of CETC was slightly decreased after RT and the gene expression increase was only moderate. This indicates that local relapse may not necessarily be directly related to CETC number changes and gene expression levels.

Two of the nine patients examined developed a second malignancy. This is a strikingly high number but attributed to a random statistical effect. The patient with the highest increase in activity of COX-2, PIK3CA, HER2, and Vimentin was diagnosed with colon cancer 84 months after treatment of breast cancer. It is unclear to what extent the CETC detected in the study are related to the development of colon cancer as EpCAM used in our method is also overexpressed in colon cancer [91]. Pat. ID 6 was diagnosed with malignant melanoma 17 month after treatment of breast cancer. This patient also showed only a moderate increase in CETC count and gene expression.

## 5. Conclusions

Procedures and methods used in this study proved to be feasible. We were able to detect changes in the CETC numbers of patients with early-stage breast cancer before radiotherapy in comparison to the end of radiotherapy.

Gene expression analysis by qRT-PCR of single CETC showed that there was an increase in the expression of selected genes involved in metastatic processes in the majority of CETC analyzed under radiotherapy. Because of the heterogeneity of results in gene expression in individual cells, further characterization of the biological and functional effects of radiotherapy is needed. The findings of this pilot study provide an important basis for a prospective follow-up study with larger numbers of patients and cells (NCT03935802).

## Figures and Tables

**Table 1:** Gene expression analysis and respective primer sequences.

Gene Name	Sequence (5′–3′) Sense (s)—Antisense (a)	Product Length Base Pairs (bp)
GAPDH	s: GAC AGT CAG CCG CAT CTT CT a: GCG CCC AAT ACG ACC AAA TC	104 bp
EpCAM	s: GGG AAA TAG CAA ATG GAC ACA a: CGA TGG AGT CCA AGT TCT GG	219 bp
NANOG	s: GGA TCC AGC TTG TCC CCA AA a: TGC ACC AGG TCT GAG TGT TC	674 bp
Bcl-2	s: TTT GTG GAA CTG TAC GGC CC a: CCG GCC AAC AAC ATG GAA AG	519 bp
TLR4	s: GGT CAG ACG GTG ATA GCG AG a: ATT AGG AAC CAC CAC GC	179 bp
COX-2	s: GAT GAT TGC CCG ACT CCC TT a: TGA AAA GGC GCA GTT TAC GC	274 bp
PIK3CA	s: CCC AGG TGG AAT GAA TGG CT a: CCA AAA GCA GGC CAA ACC TC	925 bp
HER2	s: AGG TAA CCC TGG CCC CTT T a: TTC AGC GGG TCT CCA TTG TC	539 bp
Vimentin	s: TCC GCA CAT TCG AGC AAA GA a: ATT CAA GTC TCA GCG GGC TC	161 bp
Ki-67	s: CCT CAG CAC CTG CTT GTT TG a: TCC CTG AGC AAC ACT GTC TTT	466 bp
c-Met	s: GGT CTT CAA GTA GCC AAA GCG a: TTC TTG CAG CCA AGT TGT	73 bp

**Table 2:** Patient characteristics.

Pat. ID	Age [Years]	Date of First Diagnosis	Initial Tumor Stage and Tumor Characteristics	Receptor Status of Tumor Cells	Endocrine Therapy during RT	Locoregional Recurrence after 5 Years	Distant Metastasis after 5 Years
1	60	05/2012	pT1c pN0(0/1sn) cM0 L0 V0 R0 G2	ER: 80% PR: 100% HER2: 0	Anastrozol	-	-
2	73	02/2012	pT2 pN0(0/2sn) cM0 L0 V0 R0 G2	ER: 85% PR: 85% HER2: 1+	Letrozol	-	-
3	68	03/2012	pT1c pN0(0/1sn) cM0 L0 V0 R0 G2	ER: 80% PR: 0% HER2: 2+	Letrozol	-	-
4	52	03/2012	pT1c (m) pN0(0/1sn) cM0 L0 V0 R0 G2	ER: 80% PR: 70% HER2: 1+	Tamoxifen	-	-
5	46	06/2012	pT1b(m) pN0(0/1sn) cM0 L0 V0 R0 G1	ER: 90% PR: 90% HER2: 2+	Tamoxifen	Local relapse 78 months after RT	-
6	55	04/2012	pT1b pN0(0/2 sn) cM0 L0 V0 R0 G2	ER: 90%, PR: 80% HER2: 1+	Letrozol	-	-
7	59	10/2011	pT1a+Tis pN0(0/1sn) cM0 L0 V0 R0 G1	ER: 100% PR: 100% HER2: 1+	Letrozol	-	-
8	51	11/2011	pT1c pN0(0/3 sn) cM0 L0 V0 R0 G3	ER: 0% PR: 0% HER2: 0	-	-	-
9	41	11/2011	pT2 pN0(0/1 sn) cM0 L0 V0 R0 G3	ER: 45% PR: 70% HER2: 2+	Tamoxifen	-	-

**Table 3:** Mean number of CETC.

Pat. ID	before Radiotherapy	after Radiotherapy	Quotient
	Number of Viable CETC/mL Whole Blood	Number of Viable CETC/mL Whole Blood	
1	3170	8640	2.72
2	2200	5860	2.66
3	2440	6100	2.50
4	18070	17340	0.96
5	1460	1220	0.83
6	2690	5150	1.91
7	4200	11480	2.73
8	2930	4150	1.42
9	11720	18120	1.55

**Table 4:** Copy numbers of Pat. ID 2.

Cell Number	GAPDH		EpCAM		NANOG		BCL-2	
	before RT	after RT	before RT	after RT	before RT	after RT	before RT	after RT
1	10.20	29.40	13.00	32.60	88.60	123.00	9.40	33.90
2	15.50	53.30	9.13	69.50	106.00	182.00	7.43	28.40
3	9.94	553.00	10.60	29.70	80.80	186.00	7.81	20.7
4	146.00	16.20	10.70	17.50	84.10	264.00	7.76	33.70
5	47.60	25.60	14.90	28.50	111.00	164.00	11.40	29.50
6	14.40	298.00	10.60	52.20	103.00	150.00	6.27	199.00
7	15.30	404.00	16.00	104.00	91.60	189.00	15.30	233.00
8	12.90	200.00	15.20	45.30	75.20	170.00	8.22	72.40
**Cell** **Number**	**COX-2**		**PIK3CA**		**HER2**		**Vimentin**	
	**before RT**	**after RT**	**before RT**	**after RT**	**before RT**	**after RT**	**before RT**	**after RT**
1	5.09	4.41	1.31	6.71	2.28	1.87	4.98	17.55
2	2.25	5.56	2.86	21.80	1.55	2.72	3.84	21.50
3	1.75	4.48	3.28	7.64	1.69	1.73	3.41	7.22
4	2.41	6.69	1.51	13.50	2.35	1.69	3.98	109.00
5	4.00	18.60	11.00	43.00	1.78	137.00	7.52	34.70
6	3.00	407.00	8.97	147.00	2.66	71.60	3.85	33.50
7	3.40	508.00	2.09	107.00	2.40	134.00	7.24	56.30
8	1.41	16.20	1.25	127.00	2.36	9.14	3.40	134.00
**Cell** **Number**	**c-Met**		**Ki-67**		**TLR 4**			
	**before RT**	**after RT**	**before RT**	**after RT**	**before RT**	**after RT**		
1	4.93	6.44	15.80	27.40	3.03	5.55		
2	3.24	6.42	14.10	68.80	29.80	7.27		
3	3.41	7.32	10.23	43.90	1.70	25.40		
4	3.93	4.94	16.80	30.00	4.01	24.90		
5	5.52	13.10	27.10	67.40	3.60	85.60		
6	3.85	32.20	23.90	104.00	2.30	724.00		
7	7.42	53.30	18.10	131.00	3.44	864.00		
8	3.70	19.10	17.40	87.00	1.34	93.20		

**Table 5:** Quotient (Q) of the CETC number (first row) and quotient of the copy number of genes before and after the end of irradiation.

Pat. ID	CETC Number	GAPDH	EpCAM	NANOG	BCL-2	COX-2
1	2.72 ^1^	3.97	8.12	4.26	8.79	18.67
2	2.66	5.81	3.79	1.93	8.84	41.65
3	2.50	3.59	2.31	1.36	3.00	4.69
4	0.96	1.85	0.67	10.45	0.87	0.51
5	0.83	1.12	1.56	1.65	1.63	1.58
6	1.91	1.30	1.70	1.04	1.63	2.69
7	2.73	4.70	6.01	3.93	4.41	4.90
Average	2.04	3.19	3.45	03.52	4.17	10.67
**Pat. ID**	**PIK3CA**	**HER2**	**Vimentin**	**c-Met**	**Ki-67**	**TLR 4**
1	5.74	10.46	6.59	8.03	3.96	9.31
2	14.68	21.07	10.83	3.97	4.19	37.18
3	6.47	4.65	1.27	2.25	1.14	3.27
4	0.25	0.16	0.50	0.84	2.82	0.23
5	1.17	0.98	2.26	1.95	1.24	1.82
6	1.78	2.05	1.99	4.90	1.05	1.33
7	2.40	15.16	1.57	7.47	4.28	5.16
Average	4.64	7.79	3.57	4.20	2.67	8.33

^1^ Red: Q ≥ 10, Yellow: 5 ≤ Q < 10, Green: 1 ≤ Q < 5, Blue: Q < 1.

## Data Availability

The datasets generated and/or analyzed during the study are not publicly available due to preservation of privacy but are available from the corresponding author on reasonable request.

## Glossary

cDNA	Complementary deoxyribonucleic acid
CETC	Circulating epithelial tumor cells
EpCAM	Epithelial cell adhesion molecule
FITC	Fluoroisothiocyanate
GAPDH	Glyceraldehyde 3-phosphate dehydrogenase
mRNA	Messenger ribonucleic acid
qRT-PCR	Quantitative real-time PCR
RT	Radiotherapy
